# What about Your Body Ornament? Experiences of Tattoo and Piercing among Italian Youths

**DOI:** 10.3390/ijerph182312429

**Published:** 2021-11-26

**Authors:** Francesca Gallè, Federica Valeriani, Daniela Marotta, Andrea De Giorgi, Annalisa Bargellini, Aida Bianco, Maria Eugenia Colucci, Maria Anna Coniglio, Laura Dallolio, Osvalda De Giglio, Gabriella Di Giuseppe, Giusy Diella, Pasqualina Laganà, Francesca Licata, Giorgio Liguori, Isabella Marchesi, Sofia Marini, Maria Teresa Montagna, Christian Napoli, Giovanni Battista Orsi, Cesira Pasquarella, Concetta Paola Pelullo, Luca Ricciardi, Vincenzo Romano Spica, Rossella Sacchetti, Stefano Tardivo, Licia Veronesi, Matteo Vitali, Carmela Protano

**Affiliations:** 1 Department of Movement Sciences and Wellbeing, University of Naples “Parthenope”, 80133 Naples, Italy; giorgio.liguori@uniparthenope.it; 2 Department of Movement, Human, and Health Sciences, University of Rome “Foro Italico”, 00135 Rome, Italy; vincenzo.romanospica@uniroma4.it; 3 Department of Public Health and Infectious Diseases, Sapienza University of Rome, 00185 Rome, Italy; daniela.marotta@uniroma1.it (D.M.); andrea.degiorgi@uniroma1.it (A.D.G.); giovanni.orsi@uniroma1.it (G.B.O.); matteo.vitali@uniroma1.it (M.V.); carmela.protano@uniroma1.it (C.P.); 4 Department of Biomedical, Metabolic and Neural Sciences, Section of Public Health, University of Modena and Reggio Emilia, 41125 Modena, Italy; annalisa.bargellini@unimore.it (A.B.); isabella.marchesi@unimore.it (I.M.); 5 Department of Health Sciences, School of Medicine, University of Catanzaro “Magna Græcia”, 88100 Catanzaro, Italy; a.bianco@unicz.it (A.B.); francesca.licata@studenti.unicz.it (F.L.); 6 Department of Medicine and Surgery, University of Parma, 43125 Parma, Italy; mariaeugenia.colucci@unipr.it (M.E.C.); ira.pasquarella@unipr.it (C.P.); licia.veronesi@unipr.it (L.V.); 7 Department of Medical and Surgical Sciences and Advanced Technologies “G.F. Ingrassia”, University of Catania, 95123 Catania, Italy; ma.coniglio@unict.it; 8 Department of Biomedical and Neuromotor Sciences Alma Mater Studiorum, University of Bologna, 40126 Bologna, Italy; laura.dallolio@unibo.it (L.D.); sofia.marini2@unibo.it (S.M.); 9 Department of Biomedical Sciences and Human Oncology, University of Bari “Aldo Moro”, 70124 Bari, Italy; osvalda.degiglio@uniba.it (O.D.G.); giusy.diella@uniba.it (G.D.); mariateresa.montagna@uniba.it (M.T.M.); 10 Department of Experimental Medicine, University of Campania “Luigi Vanvitelli”, 80138 Naples, Italy; gabriella.digiuseppe@unicampania.it (G.D.G.); concettapaola.pelullo@unicampania.it (C.P.P.); 11 Department of Biomedical and Dental Sciences and Morphofunctional Imaging, University of Messina, 98122 Messina, Italy; plagana@unime.it; 12 Department of Medical Surgical Sciences and Translational Medicine, Sapienza University of Rome, 00139 Rome, Italy; christian.napoli@uniroma1.it; 13 Department of Neuroscience, Mental Health and Sense Organs (NESMOS), Sapienza University of Rome, 00189 Rome, Italy; ricciardi.lu@gmail.com; 14 Department of Education Studies “Giovanni Maria Bertin”, University of Bologna, 40126 Bologna, Italy; rossella.sacchetti@unibo.it; 15 Department of Diagnostic and Public Health, University of Verona, 37134 Verona, Italy; stefano.tardivo@univr.it

**Keywords:** body piercing, tattooing, young adults, adverse effects, prevention and control

## Abstract

Background: tattooing and piercing are increasingly common, especially among youths. However, several health complications may be associated with these practices if basic hygiene rules are not respected. This multicenter study was aimed at exploring tattoo and piercing experiences reported by a large sample of Italian undergraduate students through a public health perspective. Methods: tattooed and/or pierced students attending 12 Italian universities were asked to complete a web-based questionnaire regarding their body art experience. Results: out of 1472 respondents, 833 (56.6%) were tattooed and 1009 (68.5%) were pierced. The greatest proportion of tattooed students (93.9%) got her/his first tattoo in a tattoo studio, while most of the pierced were serviced in a jewelry store (48.0%). The pierced ones were less informed on health issues related to the procedure (56.0% versus 77.8% of tattooed *p* < 0.001), and tattooists were reportedly more attentive to hygiene rules (instrument sterilization 91.5% versus 79.1% of piercers, *p* < 0.001; use of disposable gloves 98.2% versus 71% of piercers, *p* < 0.001). Conclusions: educational interventions for both professionals and communities are needed to improve the awareness and the control of health risks related to body art throughout the Italian territory.

## 1. Introduction

Nowadays, tattoos and piercings represent common types of body modification, especially among adolescents and young adults [1,2,3,4]. Although they present different sociological features, and individuals seeking a tattoo or a piercing can have different underlying motivations and encounter different purchase options [1,5], both these practices may be associated with health consequences. Tattooing consists in the introduction of exogenous pigments into the dermis, resulting in a permanent design [4]. Piercing is made by creating openings through the skin or cartilage to insert decorative ornaments, such as rings, studs, or pins generally fabricated of stainless steel, titanium, gold, niobium, or acrylic [5]. Therefore, both these procedures require that sharpened tools pass through the skin/mucous barrier, which may lead to the penetration of infectious agents coming from skin, pigments, or instruments in the underlying tissues [1,3,6,7,8,9]. Bacterial local (e.g., abscesses, necrotizing fasciitis) and systemic infections (e.g., endocarditis, septic shock), so as the transmission of cutaneous and bloodborne viruses (human papillomavirus, herpes simplex virus, hepatitis B, hepatitis C, and human immunodeficiency viruses) were associated with tattooing [10,11]. As for body piercing, the greater tissue damage and presence of a foreign body, with a consequently longer healing time of the wound, make bacterial infections of the skin and soft tissues common consequences of this practice; the risk of viral hepatitis transmission was also reported [12].

In addition, hypersensitivity and allergic reactions to metals, inks, local anesthetic, and antiseptic creams employed during tattooing and piercing are common [7,8,9,13]. Nonallergic inflammatory reactions (for example, cutaneous granuloma and pseudolymphoma) as well as allergic reactions occurring during or after wound healing were associated with tattooing [13], and allergic contact dermatitis caused by metal allergens is a usual reaction to body piercing [14].

The risk of developing a subsequent infection or immune reaction is related to the body site involved, the immune status of the customer, the adoption of hygienic rules during and after the procedure, and the experience of the tattooist/piercer [7,8,9]. Licensed operators, in fact, should have adequate education on possible health risks of these procedures and must follow strict control measures, such as the use of single use inks and sterile needles and jewels. Furthermore, they should identify among their customers those presenting possible contraindications or needing special precautions, inform their clients of possible consequences of tattoos and piercings, and recommend aftercare procedures to prevent complications [15]. For example, people with underlying health issues that may imply prolonged wound healing, weak immune response to pathogens, or immune overreaction should be warned about their increased risk of complications, while individuals undergoing surgical interventions, such as pregnant women or women who are considering a pregnancy within one year, should not acquire body modifications [14,15].

In Italy, the local regulations establish that piercing or tattoo should be performed after obtaining the subject’s written consent to undergo the specific treatment, declaring his or her awareness regarding the obtained information. If the person who requests a tattoo or piercing is a minor, parental consent based on the provision of exhaustive information about procedure, risks, and precautions to be taken after the body modification and possible subsequent removal is needed [16]. However, tattoos/piercings may be performed in nonprofessional parlors by unlicensed personnel, using nonsterile and low-quality equipment [17]. Customers, especially youths, may not be aware of health risks related to these procedures and cannot be able to identify licensed artists or to assess whether the tattooist/piercer is using adequate control measures [2,13]. Several investigations were performed at local level in Italy to evaluate body art experience of youths, to assess the spread of this phenomenon, explore their motivation, and evaluate the adoption of control measures during the procedures they underwent [18,19,20,21]. This multicenter study was aimed at examining tattoo and piercing experiences reported by a large sample of Italian undergraduate students, to draw a nationwide picture of these practices and to assess the adoption of control measures in Italian tattoo/piercing parlors.

## 2. Materials and Methods

### 2.1. Study Design and Participants

A cross-sectional study named “Study on Undergraduate Perception of Risks of Body Art” (SUPeRBA) was performed between April 2020 and January 2021 for evaluating the knowledge and the awareness of health risks associated to body arts. For this purpose, a web-based questionnaire was administered to a large sample of undergraduate students from twelve selected public Italian universities [22]. The present study was carried out considering just the subpopulation of participants who had at least one tattoo or one piercing to trace a picture of this community and to study in depth some features of body art practices. 

With a total population of about 500,000 undergraduate students attending the participant universities, a sample size of at least 246 tattooed and 323 pierced students were reached, with a prevalence of about 20% and 30% of tattooing and piercing, respectively, among university students in Italy [22], and a confidence interval of 95%. 

The study was carried out according to the principles of the Declaration of Helsinki. Ethical approval was obtained from the Research Committee of the University of Rome “Foro Italico” (approval n CAR 31/2020) and from the academic deans.

### 2.2. Questionnaire

A structured anonymous questionnaire based on that used in our previous investigations was used [18,19,20,21]. The questionnaire was described previously, here we report a brief description of the questions used for collecting the information investigated in the present study. The first section of the questionnaire was focused on socio-demographic information of tattooed/pierced participants: the type of body art they have (tattoo/piercing/both), their gender, age, university, degree course and year attended, nationality (Italian/other), residential status (residing in the area of the university/commuting/not residing but living in the area of the university), educational level of parents (primary school/middle school/high school/degree or postdegree), and whether their parents have a tattoo or a piercing. The second section investigated the students’ experience regarding body art. Participants were asked to report how many tattoos/piercings they had, when they got their first tattoo/piercing and in which part of their body (head/neck, trunk, or arms/legs), and where they got them (home, street tattoo artists, beauticians, or ink studios for tattoos; home, jewelries, beauticians, or piercing studios for piercings). Finally, we asked if they had any complications after their first tattoo or piercing. In the last section, the questionnaire examined under what conditions they experienced the body modification: if their parents know about their tattoo or piercing (if they were minor); if written consent was involved; if they were advised about risks of these practices and who informed them; if sterile instruments and disposable gloves were used by practitioner, and if they received information about tattoo or piercing care. 

The study took place during the COVID-19 epidemic. To control the transmission of the disease, during that period all the universities involved in the study provided online courses, even together with those in person. The questionnaire and the aims of the study were presented to the undergraduates during the lessons and administered through the Google modules platform by sending them a link to the questionnaire.

### 2.3. Statistical Analysis

A descriptive analysis was performed on sociodemographic characteristics and answers of participants. Continuous variables were expressed as mean values ± standard deviation (SD), while categorical variables were reported as number and percentage values of respondents. Univariate analyses were performed using the chi-squared test (with Yates correction) to assess possible differences in sociodemographic characteristics of tattooed, pierced and tattooed/pierced students, and to explore the differences in body art procedure experienced by tattooed and pierced students. 

A logistic regression analysis was performed by considering for both tattooed and pierced subjects the choice of a professional operator as an outcome and all the sociodemographic features of participants (age lower or equal/higher than median value, female or male gender, Italian or other nationality, north/center or south Italy university, life science or other degree course, residing/commuting or living in the area, parents’ school or university educational level, and parents with tattoo/piercing) as independent variables. Significant associations were reported as odds ratios and corresponding 95% confidence interval (OR 95%CI).

The significance level was assumed as *p* < 0.05. Analyses were conducted using the IBM SPSS Statistics for Windows, version 27.0 (IBM Corp.: Armonk, NY, USA).

## 3. Results

On a total of 2985 undergraduates who participated in the SUPeRBA study, 833 (27.9%) had at least a tattoo and 1009 (33.8%) had a piercing. In total, 1472 completed the section of the questionnaire regarding their body art experience and their answers were included in the analysis. Table 1 shows the main characteristics of participants grouped on the basis of the type of body ornament they reported, with corresponding *p* values related to the differences found among groups: those who had one or more tattoos, those who had just one or more piercings and those who had both tattoo/s and piercing/s.

In total, 31.5% of the participants had at least one tattoo, 43.5% had at least one piercing and 25.1% had both tattoo and piercing. Participants had an average age of about 23 years, were predominantly females and Italian, and came mostly by life science courses. The educational levels of mother and father were “High school” for both parents and about one third of tattooed or tattooed and pierced participants had a parent with a tattoo, while less than 10% of those who had a body ornament had a parent with a piercing. Table 2 summarizes the main information related to tattooing and body piercing experience of all the participants, including those having just one or more tattoos, those having just one or more piercing and those having both tattoo/s and piercing/s.

The mean number of tattoos and piercing was about 3 and 2.5, respectively. The first tattoo was completed at the age of 19 years and principally performed on arms or legs, while the first piercing was completed earlier, at the age of 16 years and mainly made on head or neck. The greatest proportion of the tattooed (93.9%) had the first tattoo done in a tattoo studio, while most of the pierced had the first piercing done in a jewelry store or in a piercing studio (48.0 and 45.7% respectively). Notice that a group of participants, even if small, stated that they had the first body art at home (4.3% of tattooed and 3.6% of pierced, respectively). Regarding the complications, just a little proportion of tattooed had a complication after the first tattoo, while more than a third of subjects with a piercing reported an adverse effect due to the practice of the first piercing. 

Table 3 shows the results of the univariate analysis on the experienced procedures of tattooing and body piercing, considering participants who had only one or more tattoos or one or more piercing.

Significant differences between the groups of tattooed and pierced students were recovered for having signed the informed consent before the practice of the body art and having received information about the risks associated with the procedure and the care of the tattoo or piercing: pierced seems to be less informed respect to tattooed on all the aspect linked to the procedure. Similarly, a significantly higher percentage of tattooed stated that the instruments used for the procedure were sterilized and that the tattooist used disposable gloves.

As for the logistic regression analysis, no variables were found to be related with the choice of nonprofessional tattooists, while attending a university from north/center Italy was less associated with the choice of nonprofessional piercers (OR 0.645, CI95% 0.493–0.844, *p* = 0.001).

## 4. Discussion

This study was aimed to profile body art practice among Italian youths, investigating both socio-demographic and health-related aspects associated with this growing phenomenon.

As for the spread of these practices, the results show an increase in the proportion of tattooed and pierced undergraduates with respect to previous studies performed in Italy about a decade ago [18,19,20,21]. This is in line also with the higher proportions of tattooed and pierced students attending the first academic years found in the sample, which testifies the increasing diffusion of this fashion among the new generations, according to the trend we previously identified [19]. Furthermore, it seems that having a tattoo or a piercing is related with other sociodemographic factors and with parental model. In particular, the results show that female gender and attending universities of southern Italy were the characteristics mainly showed by tattooed and pierced undergraduates. The result related with gender difference is partially in contrast with those of Lahousen et al., who found a higher prevalence of tattoos in men and piercing in women in Germany [23]. As for the geographical area, the percentages of tattooed and pierced students obtained in this study are even higher than those reported in the previous studies performed in two regions of southern Italy [18,19,20,21]. In addition, tattooing practice seems to be related with having a parent who is tattooed, which is in line with previous evidence [24].

Regarding the body art experienced, the age at first piercing was lower than that referred for the first tattoo, as previously reported [21]. As such, while presenting the questionnaire, we asked participants to avoid reporting information about possible earlobe piercings practiced in their childhood and decided by their parents. Therefore, it is presumable that our finding reflects the reality regarding the choices of young Italians.

Moreover, it is remarkable that a professional studio was reported as the location of the practice by the majority of tattooed students and by less than the half of those who had a piercing. This can be in part explained by the high proportion of piercings performed in a jewelry store, which is a common choice in Italy. In addition, around 4% of reported tattoos and piercings were practiced at home. Although the proportions of students who received their body ornament in professional parlors were notably higher than that we previously registered [18,19,20,21], this aspect is of relevant concern for public health and should be carefully considered due to the scarce possibility to control health risks in nonprofessional settings.

Furthermore, a higher number of complications was reported for piercings than for tattoos, which may probably be related with the location chosen. It is presumable that body art professionals may adequately address their customers to avoid complications by giving them fundamental aftercare indications. With regard to this, a lower percentage of participants in this study who received aftercare information was found among pierced students than in tattooed group.

As for health risk awareness, on the total of students who were previously informed about health risks, the majority reported the operator as the main source of information, both for tattooed and pierced groups. However, the great proportion of students who reported no information is worrying and highlights the need of further efforts in this direction.

With regard to the choice of the operator, in the logistic regression analysis, addressing themselves to professional piercers was found to be more common among students from northern or central Italy, which is in line with the better knowledge of piercing risks shown by these student populations in our previous survey [22].

Furthermore, the notable proportion of students who did not inform their parents about their body modification and who did not report the signature of an informed consent represent other critical issues, especially for piercing. Similarly, those who underwent a piercing referred less the use of sterilized instruments and gloves by the operator.

These results can probably be explained by regional differences. In 1998, the Italian Ministry of Health issued the “Guidelines for the implementation of procedures for tattooing and piercing in safe conditions and the subsequent interpretative letter no. 2.8/633, which were focused on the risk of infection and the toxic effects due to substances used for tattoos and indicated the basic rules for hygiene and environmental control of these risks [25]. The Ministry requirements for a body art professional are to be at least 18 years old and the possession of a certificate of participation in a training course for tattoo artists. The public health departments of the Italian regions were allowed to adopt the guidelines with modifications and the consequent promulgation of regional directives and/or other legislative measures. Most of the regions simply enforced the Ministry guidelines, a few issued more stringent rules, and others had no regulations at all [26]. The qualification of tattoo artists varies at the regional level, with the length of courses ranging from 14 to 600 h. This poses serious issues in professionals’ education towards health risks and the consequent adoption of appropriate control measures. Consequently, the level of health protection for customers varies across the different regions. This could have determined the detection of those subgroups of participants who reported unhealthy operator’s deeds during their experience.

This study has some limitations. Firstly, as stated before, the sample was not built randomly and did not include students from all the Italian regions, which limits its representativeness. Furthermore, only university students were enrolled in this study, and only those who attended the lessons were invited to take part in the study. Therefore, the reported findings cannot pertain the body art experiences of all the Italian young adults. Moreover, since this investigation was aimed at assessing the physical consequences of body ornament on health, we did not analyze motivations or expectations towards the modification gotten, nor psychological or behavioral aspects related to the choices made by participants. Further studies may be useful to investigate these items, also in nonundergraduate individuals and in other age classes.

However, this survey analyzed, for the first time at a national level, tattoo and piercing experiences of Italian youths through a public health perspective. This represents the main strength of this study.

## 5. Conclusions

The findings of this study highlight some critical issues related to tattoo and piercing practice in Italy. A high proportion of participants presented themselves to operators different from professional tattooists and piercers. This may have consequences on customers’ awareness of health risks and body ornament aftercare, with related complications. Furthermore, it seems that not all the operators follow the main recommended measures of risk control, nor provide informed consent to their clients. Public health policies should address these points through the enhancement of educational interventions for both professionals and community. 

## Figures and Tables

**Table 1:** Sociodemographic characteristics of participants (*n* = 1472) grouped for type of body art gotten.

Variables		Tattooed (*n* = 463)		Pierced (*n* = 639)		Tattooed and Pierced (*n* = 370)		*p* Value ^†^
Age (mean ± SD)		23.3 ± 3.9		22.8 ± 3.9		23.6 ± 4.2		0.007
		***n***	**%**	***n***	**%**	***n***	**%**	
Gender	Female	368	79.5	498	77.9	330	89.2	<0.001
	Male	95	20.5	141	22.1	40	10.8	
Participant’s Nationality	Italian	453	97.8	618	96.7	362	97.8	0.411
	Other	10	2.2	21	3.3	8	2.2	
University location	North	143	30.9	189	29.6	101	27.3	0.003
	Centre	116	25.1	200	31.3	140	37.8	
	South	204	44.1	250	39.1	129	34.9	
Type of course	Life science	358	77.3	514	80.4	289	78.1	0.419
	Other	105	22.7	125	19.6	81	21.9	
Year of study course	First year	106	22.9	152	23.8	68	18.4	0.014
	Second year	120	25.9	145	22.7	91	24.6	
	Third year	117	25.3	171	26.8	100	27.0	
	Fourth year	34	7.3	30	4.7	12	3.2	
	Fifth year	32	6.9	62	9.7	35	9.5	
	Sixth year	19	4.1	44	6.9	31	8.4	
	Outside prescribed time	35	7.6	35	5.5	33	8.9	
Residential status	Residing in the area	149	32.2	177	27.7	121	32.7	0.154
	Commuting	184	39.7	265	41.5	129	34.9	
	Not residing but living in the area	130	28.1	197	30.8	120	32.4	
Father’s educational level	Primary school	16	3.5	15	2.3	8	2.2	0.290
	Middle school	121	26.1	166	26.0	104	28.1	
	High school	226	48.8	290	45.4	182	49.2	
	Degree or postdegree	100	21.6	168	26.3	76	20.5	
Mother’s educational level	Primary school	13	2.8	19	3.0	11	3.0	0.745
	Middle school	91	19.7	114	17.8	82	22.2	
	High school	243	52.5	335	52.4	179	48.4	
	Degree or postdegree	116	25.1	171	26.8	98	26.5	
Does your mother/father have a tattoo?	Yes	150	32.4	55	8.6	110	29.7	<0.001
	No	313	67.6	584	91.4	260	70.3	
Does your mother/father have a piercing?	Yes	32	6.9	46	7.2	33	8.9	0.502
	No	431	93.1	593	92.8	337	91.1	

**^†^** chi-squared test.

**Table 2:** Information related to tattooing and body piercing experience of participants (tattooed *n* = 833; pierced *n* = 1009).

Variables	Mean Values ± SD	Participants	
		*n*	%
How many tattoos do you have?	3 ± 3.1		
How old were you when you got your first tattoo?	19 ± 2.9		
Which part of your body did you choose for your first tattoo?			
Head/Neck		45	5.4
Trunk		307	36.9
Arms/Legs		479	57.6
Where did you get your first tattoo?			
Home		36	4.3
Street tattoo artist		11	1.3
Beautician		4	0.5
Ink studio		779	93.9
Did you have any complications after you first tattoo?			
Yes		17	2.1
No		812	97.9
How many piercings do you have?	2.5 ± 2.2		
How old were you when you got your first piercing?	16.3 ± 4.0		
Which part of your body did you choose for your first piercing?			
Head/Neck		855	85.2
Trunk		142	14.2
Arms/Legs		6	0.6
Where did you get your first piercing?			
Home		36	3.6
Jewelry store		474	48.0
Beautician		26	2.3
Piercing studio		451	45.7
Did you have any complications after you first piercing?			
Yes		335	33.6
No		661	66.4

**Table 3:** Answers related to tattooing or body piercing procedure experienced by participants (*n* = 1102).

Variable		Tattooed (*n* = 463)		Pierced (*n* = 639)		*p* Value ^†^
		*n*	%	*n*	%	
Did your parents know about your tattoo/piercing if you were a minor when you got it?	Yes	74	87.1	374	84.0	0.623
	No	11	12.9	71	16.0	
	Missing	378		194		
Did you or your parents sign a written consent?	Yes	96	65.3	266	49.5	0.001
	No	51	34.7	271	50.5	
	Missing	316		102		
Have you been informed about the risks of these practices before getting your tattoo/piercing?	Yes	165	77.8	355	56.0	<0.001
	No	47	22.2	279	44.0	
	Missing	251		5		
Who informed you about the risks?	Tattoo artist/piercer with written consent	97	58.8	196	55.4	0.762
	Tattoo artist/piercer with spoken consent	40	24.2	94	26.6	
	Other	28	17.0	64	18.1	
	Missing	298		285		
When you got your tattoo/piercing, were the instruments sterilized?	Yes	205	91.5	499	79.1	<0.001
	No	1	0.5	36	5.7	
	I do not know	18	8.0	96	15.2	
	Missing	239		8		
When you got your tattoo/piercing, did the tattooist/piercer use disposable gloves?	Yes	217	98.2	446	71.0	<0.001
	No	0	0.0	85	13.5	
	I do not know	4	1.8	97	15.5	
	Missing	242		11		
When you got your tattoo/piercing, did you get informed about the care of your tattoo/piercing?	Yes	223	98.7	595	93.6	0.009
	No	3	1.3	30	4.7	
	I do not know	0	0.0	11	1.7	
	Missing	237		3		

**^†^** chi-squared test.

## Data Availability

Not applicable.

## References

[1] Stirn A. (2003). Body piercing: Medical consequences and psychological motivations. Lancet.

[2] Kluger N., Serup J., Kluger N., Bäumler W. (2015). Epidemiology of tattoos in industrialized countries. Tattooed Skin and Health.

[3] Islam P.S., Chang C., Selmi C., Generali E., Huntley A., Teuber S.S., Gershwin M.E. (2016). Medical Complications of tattoos: A comprehensive review. Clin. Rev. Allergy Immunol..

[4] Barnett J. (2003). Health implications of body piercing and tattooing: A literature review. Nurs. Times.

[5] Raggiotto F., Mason M.C., Moretti A. (2018). A lens of analysis for consumption practices in tattooing. Micro Macro Mark..

[6] Giulbudagian M., Schreiver I., Singh A.V., Laux P., Luch A. (2020). Safety of tattoos and permanent make-up: A regulatory view. Arch. Toxicol..

[7] Holbrook J., Minocha J., Laumann A. (2012). Body piercing: Complications and prevention of health risks. Am. J. Clin. Dermatol..

[8] Laux P., Tralau T., Tentschert J., Blume A., Al Dahouk S., Bäumler W., Bernstein E., Bocca B., Alimonti A., Colebrook H. (2016). A medical-toxicological view of tattooing. Lancet.

[9] Sindoni A., Valeriani F., Gallè F., Liguori G., Spica V.R., Vitali M., Protano C. (2021). Adverse effects related to tattoos in the community setting: A systematic review. J. Epidemiol. Community Health.

[10] Dieckmann R., Boone I., Brockmann S.O., Hammerl J.A., Kolb-Mäurer A., Goebeler M., Luch A., Al Dahouk S. (2016). The Risk of Bacterial Infection After Tattooing. Dtsch. Arztebl. Int..

[11] Cohen P.R. (2021). Tattoo-Associated Viral Infections: A Review. Clin. Cosmet. Investig. Dermatol..

[12] Yang S., Wang D., Zhang Y., Yu C., Ren J., Xu K., Deng M., Tian G., Ding C., Cao Q. (2015). Transmission of Hepatitis B and C Virus Infection Through Body Piercing: A Systematic Review and Meta-Analysis. Medicine.

[13] Weiß K.T., Schreiver I., Siewert K., Luch A., Haslböck B., Berneburg M., Bäumler W. (2021). Tattoos-more than just colored skin? Searching for tattoo allergens. J. Dtsch. Dermatol. Ges..

[14] Van Hoover C., Rademayer C.A., Farley C.L. (2017). Body Piercing: Motivations and Implications for Health. J. Midwifery Women’s Health.

[15] De Cuyper C. (2020). How to advise a patient who wants a tattoo?. Presse Med..

[16] Conti A., Bin P., Casella C., Capasso E., Fedeli P., Salzano F.A., Terracciano L., Piras M. (2018). Piercing and Tattoos in Adolescents: Legal and Medico-legal Implications. Open Med..

[17] Patel M., Cobbs C.G. (2015). Infections from body piercing and tattoos. Microbiol. Spectr..

[18] Cegolon L., Miatto E., Bortolotto M., Benetton M., Mazzoleni F., Mastrangelo G. (2010). Body piercing and tattoo: Awareness of health related risks among 4,277 Italian secondary school adolescents. BMC Public Health.

[19] Gallè F., Mancusi C., Di Onofrio V., Visciano A., Alfano V., Mastronuzzi R., Guida M., Liguori G. (2011). Awareness of health risks related to body art practices among youth in Naples, Italy: A descriptive convenience sample study. BMC Public Health.

[20] Quaranta A., Napoli C., Fasano F., Montagna C., Caggiano G., Montagna M.T. (2011). Body piercing and tattoos: A survey on young adults’ knowledge of the risks and practices in body art. BMC Public Health.

[21] Gallè F., Quaranta A., Napoli C., Di Onofrio V., Alfano V., Montagna M.T., Liguori G. (2012). Body art practices and health risks: Young adults’ knowledge in two regions of southern Italy. Ann. Ig..

[22] Protano C., Valeriani F., Marotta D., Bargellini A., Bianco A., Caggiano G., Colucci M.E., Coniglio M.A., Dallolio L., De Giglio O. (2021). Assessing Undergraduates’ Perception of Risks Related to Body Art in Italy: The SUPeRBA Multicenter Cross-Sectional Study. Int. J. Environ. Res. Public Health.

[23] Lahousen T., Linder M.D., Gieler U., Hofmeister D., Trapp E.M., Borkenhagen A., Kapfhammer H.P., Brähler E. (2019). Body modification in Germany: Prevalence, gender differences and attitude towards cosmetic surgery. G. Ital. Dermatol. Venereol..

[24] Roberts A.E., Koch J.R., Armstrong M.L., Owen D.C. (2006). Correlates of tattoos and reference groups. Psychol. Rep..

[25] Ministero della Sanità (1998). Linee Guida del Ministero Della Sanità per l’Esecuzione di Procedure di Tatuaggio e Piercing in Condizioni di Sicurezza. Circolare 5 Febbraio 1998 n. 2.9/156.

[26] Renzoni A., Pirrera A., Novello F., Diamante M.S., Guarino C. (2015). Implementation of European Council resolution ResAP(2008)1 in Italy. National and regional regulation of tattoo practices: Diversity and challenges. Curr. Probl. Dermatol..

